# Comparative *in vitro *study of the antimicrobial activities of different commercial antibiotic products for intravenous administration

**DOI:** 10.1186/1472-6904-10-3

**Published:** 2010-01-29

**Authors:** Edelberto Silva, Jorge A Díaz, María J Arias, Angela P Hernández, Andrés de la Torre

**Affiliations:** 1 UNIVERSIDAD NACIONAL DE COLOMBIA, FACULTAD DE CIENCIAS, DEPARTAMENTO DE FARMACIA, Laboratorio de Asesorías e Investigaciones en Microbiología. Postal Code: 472. Ciudad Universitaria. Carrera 30 Calle 45. A.A. 14490, Bogotá D. C., Colombia

## Abstract

**Background:**

The antimicrobial resistance is a global problem, probably due to the indiscriminate and irrational use of antibiotics, prescriptions for incorrect medicines or incorrect determinations of dose, route and/or duration. Another consideration is the uncertainty of patients receiving antibiotics about whether the quality of a generic medicine is equal to, greater than or less than its equivalent brand-name drug. The antibiotics behaviors must be evaluated *in vitro *and *in vivo *in order to confirm their suitability for therapeutic use.

**Methods:**

The antimicrobial activities of Meropenem and Piperacillin/Tazobactam were studied by microbiological assays to determine their potencies (content), minimal inhibitory concentrations (MICs), critical concentrations and capacity to produce spontaneous drug-resistant mutants.

**Results:**

With respect to potency (content) all the products fulfill USP requirements, so they should all be considered pharmaceutical equivalents. The MIC values of the samples evaluated (trade marks and generics) were the same for each strain tested, indicating that all products behaved similarly. The critical concentration values were very similar for all samples, and the ratios between the critical concentration of the standard and those of each sample were similar to the ratios of their specific antibiotic contents. Overall, therefore, the results showed no significant differences among samples. Finally, the production of spontaneous mutants did not differ significantly among the samples evaluated.

**Conclusions:**

All the samples are pharmaceutical equivalents and the products can be used in antimicrobial therapy.

## Background

In the past few decades, antimicrobial resistance has been seen globally in several pathogenic microorganisms, including some that were antibiotic-sensitive until recently. This is probably due to the indiscriminate and irrational use of these medicines [1,2]. According to the World Health Organization [1], the increase in antimicrobial resistance stems from a number of factors, including lack of knowledge about it among prescribing doctors, which leads to unnecessary prescriptions. Inadequate diagnosis or lack of diagnosis also leads to the use of antimicrobials against a "possible infection". Also, prescriptions for incorrect medicines or incorrect determinations of dose, route and/or duration of treatment often occur in response to pressure from companies or patients and the desire for profit. It has been recognized that medical visitors or commercially oriented publications are the main sources of information about medicines.

Another important consideration is the uncertainty of patients receiving antibiotics about whether the quality of a generic medicine is equal to, greater than or less than its equivalent brand-name drug. The belief that "the more expensive the product, the more effective" is shared by some doctors and pharmacists. This misconception leads to unnecessary use of the newest antibiotics simply because they are more expensive and broad-spectrum, and this in turn promotes the selection of microorganisms resistant to them, increasing expense without real benefit [1]. Within this myth of "the more expensive, the more effective" we can include innovative products and generic ones because the public tends to believe that the generic product, because of its low price, is of bad quality, and therefore ineffective. For all medicines (generic or brand-name), especially antibiotics, effectiveness and safety are vital qualities; without them, the health of the patient is at risk. To remove any doubts about antibiotics, we must evaluate their behaviors *in vitro *and *in vivo *in order to confirm their suitability for therapeutic use.

In order to dispel doubts about the efficacy of generic antibiotics, arising from complaints from the medical community and reported in the literature and at international meetings [3-5], our group decided to conduct a broad-based study focusing on the quality and effectiveness of commercial antibiotics in our country (Colombia). We have already studied the effectiveness of some compounds using biological assays with microorganisms: Ceftriaxone, Cefotaxime, Ceftazidime, Ampicillin/Sulbactam and Imipenem/Cilastatin. The results have shown that experimental, brand-name and generic products are pharmaceutical equivalents; they all fulfill the requirements of the U. S. Pharmacopoeia (XXVIII) in relation to their activities [6].

In this paper, we present a comparative study of Meropenem and Piperacillin/Tazobactam using commercial products (experimental, brand-name and generic). We evaluated their antimicrobial activities by determining their potencies, critical concentrations, minimal inhibitory concentrations and minimal lethal concentrations. In respect of these parameters, all the products meet the proper standards of quality for pharmaceutical products, so we conclude that they all exhibit the same level of antimicrobial activity.

## Methods

### Microorganisms

To validate the microbiological assay for evaluating the potencies of Meropenem and Piperacillin, we used *Bacillus subtilis *ATCC 6633, *Staphylococcus aureus *ATCC 29737 and ATCC 6538p, *Pseudomonas aeruginosa *ATCC 25619 and ATCC 9027, *Micrococcus luteus *ATCC 9341, *Escherichia coli *ATCC 10536, *Klebsiella pneumoniae *ATCC 10031 and *Streptococcus faecalis*. For MIC and MLC studies we used *Acinetobacter baumanii *strains 59, 139, 147 and 173, vancomycin-resistant *Enterococcus gallinarum*, *Streptococcus faecalis *ATCC 29212, a nosocomial strain 319623 and a vancomycin-sensitive strain, *Escherichia coli *strains 39, 50 and 69, *Klebsiella pneumoniae *strains 1, 43, 63, 65 and 207, *Pseudomonas aeruginosa *strains 42, 74, 151 and 157, and *Staphylococcus aureus *strains 287, 291 and ATCC 25923. All microorganisms were grown in Mueller Hinton (MH) broth (incubated at 35°C for 24 h). Each strain was then plated on MH agar to obtain isolated colonies, which were then used to make larger cultures in MH medium. The cultures were harvested with cryopreservation broth. A portion of each was kept in a cryovial at -70°C, while the other portion was used to prepare a suspension with 25% transmittance at 600 nm (25%T) to develop *in vitro *assays. These suspensions were kept in cryovials at -70°C.

### Analytical Bioassay

This was established and validated for Meropenem and Piperacillin/Tazobactam. First, the most appropriate microorganisms were selected, the proper concentration range was determined, and the linearity, precision, specificity and stability of the compound in question were assessed [6]. All samples were then evaluated with the analytical bioassay under the chosen conditions.

### Minimal Inhibitory Concentration (MIC) and Minimal Lethal Concentration (MLC)

Assays to assess these parameters were developed in two parts. (1) **Preparation of inocula: **the number of colony forming units (CFU) was determined for each suspension at 25%T in order to prepare inocula of 1-5 × 10^6 ^CFU/ml. (2) **MIC and MLC determination by micro-dilution: **samples were diluted to 2 mg/ml for evaluation. Using a multichannel pipette, 100 μl of antibiotic sample was placed in each well of a 96-well ELISA plate, with 200 μl in column 12. Next, 100 μl of the antibiotic solution (2 mg/ml) was placed in the first column and thoroughly mixed by pipetting. From these wells, 100 μl was added to the second column and mixed, and this procedure was repeated up to column 10, after which the 100-μl portion was discarded. Columns 11 and 12 were positive and negative controls, respectively. Each row (A to H) represented a different sample to be analyzed. Each inoculum (100 μl) was then pipetted into each microplate, which was incubated at 37°C for 24 h. Growth in the wells was assessed. The lowest dilution showing no growth, the first dilution with growth, and the two controls were plated on to MH agar. The **MIC **was defined as the lowest dilution that showed no growth on the ELISA plate but showed growth on MH agar; the **MLC **was defined as the lowest dilution that did not show growth on either the ELISA plate or MH agar [7].

### Critical Concentration (CC)

The CC was determined similarly to the analytical bioassay. The inocula for **MIC **and **MLC **determination and serial two-fold dilutions of each sample from 500 to 0.244 μg/ml were used. The halo of inhibition was measured, and the crown length X was calculated (inhibition halo diameter minus reservoir diameter divided by 2). Log concentration vs. X^2 ^was plotted, and linear regression (*y = mx + b*) was applied. The y-intercept (*b*) is equivalent to the log of the CC [7].

### Spontaneous mutants

Spontaneous mutation was analyzed similarly to the analytical bioassay. Again, the inocula for **MIC **and **MLC **determination were applied. Specific microorganisms and dilutions were selected after determination of critical concentrations. On each plate, a dilution of the USP standard and samples of the same concentration were used.

### Samples

Commercial products purchased from the pharmacies of different hospitals in Bogotá, D. C. Colombia, were analyzed. They included innovator (MERONEM^®^, TAZOCIN^®^), trademark products and generic products of Meropenem and Piperacillin/Tazobactam. All the samples declared the contents were 1 g. They were all diluted in sterile water in 100 ml volumetric flasks. The solutions were divided into 5 ml fractions for storage at -70°C; they were diluted to 1 mg/ml to develop the analytical bioassays and to 2 mg/ml for MIC and MLC assays.

## Results

### Analytical Bioassay

#### Microorganism Selection

**Figure 1:**
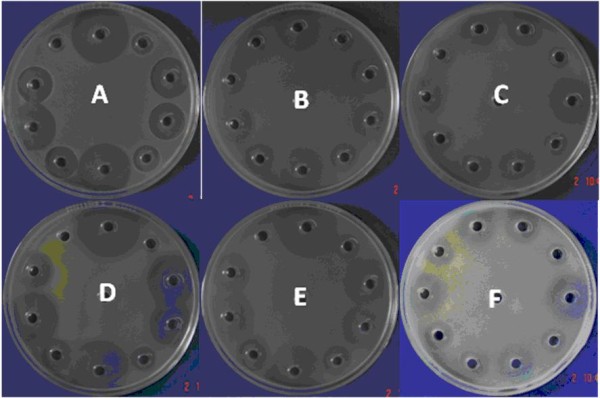
**Bioassay of Meropenem (USP standard) against (A) *Bacillus subtilis *ATCC 6633, (B) *Escherichia coli *ATCC 10536, (C) *Klebsiella pneumoniae *ATCC 10031, (D) *Micrococcus luteus *ATCC 9341, (E) *Pseudomonas aeruginosa *ATCC 25619 and (F) *Staphylococcus aureus *ATCC 29737**.

**Figure 2:**
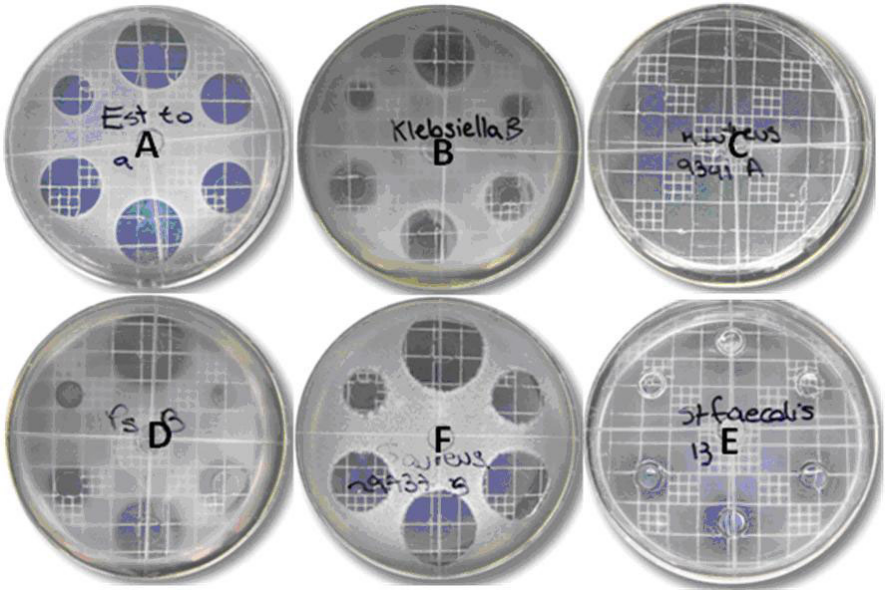
**Bioassay of Piperacillin (USP standard)/Tazobactam against (A) *Bacillus subtilis *ATCC 6633, (B) *Klebsiella pneumoniae *ATCC 10031, (C) *Micrococcus luteus *ATCC 9341, (D) *Pseudomonas aeruginosa *ATCC 25619, (E) *Staphylococcus aureus *ATCC 29737 and (F) *Streptococcus faecalis***. Cited on page 6.

The selection criteria were well-defined edges on inhibition haloes, halo diameters no greater than 30 to 35 mm, and no generation of spontaneous mutants under antibiotic treatment [8,9]. Figures 1 and 2 show the responses of various microorganisms to Meropenem and Piperacillin/Tazobactam, respectively. On the basis of these results, *B. subtilis *ATCC 6633 was selected for further experiments because it fulfilled all the criteria when treated with Meropenem and Piperacillin/Tazobactam.

#### Determination of culture medium pH, incubation time and concentration range

The results for Meropenem showed that pH 8 is optimal because the inhibition haloes were well defined at that pH. In the case of Piperacillin/Tazobactam, the optimum pH was between 6.0 and 6.5. Therefore, the assay for Meropenem is best carried out in antibiotic medium number 11 and the assay for Piperacillin/Tazobactam in antibiotic medium number 1.

The assays for both antibiotics require between 8 and 10 h incubation. This is less than many common assays, which require between 18 and 24 h.

**Figure 3:**
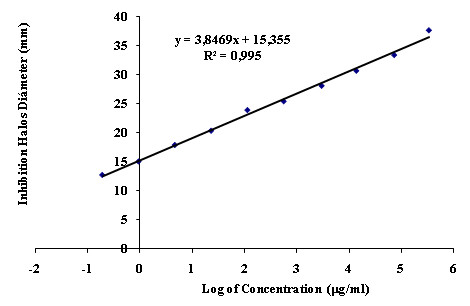
**Calibration curve of ten concentrations of Meropenem to determine the optimal test range**.

Ten concentrations were used (two-fold dilutions from 250 to 0.488 μg/ml for Meropenem and from 1000 to 1.9531 μg/ml for Piperacillin/Tazobactam). Table 1 shows that linearity was best in the range between C5 and C9 (15.62 to 0.9976 μg/ml) (R^2 ^= 0.9996). For practical reasons, however, the range was adjusted to 25 μg/ml to 1.5625 μg/ml, which also showed good linearity (R^2 ^= 0.9996, Figure 3).

**Table 1:** Evaluation of the range of concentrations for Meropenem (USP standard)

Concentration Range		Equation		
**From**	**To**	**Slope**	**Intercept**	**R^2^**

**C1**	C9	3.8469	15.355	0.995
**C1**	C5	4.288	13.36	0.9861
**C2**	C6	3.4631	16.437	0.9911
**C3**	C7	3.5659	15.967	0.9881
**C4**	C8	3.6377	15.735	0.9878
**C5**	**C9**	**3.8575**	**15.374**	**0.9976**
**C6**	C10	3.9901	15.42	0.9945

**Figure 4:**
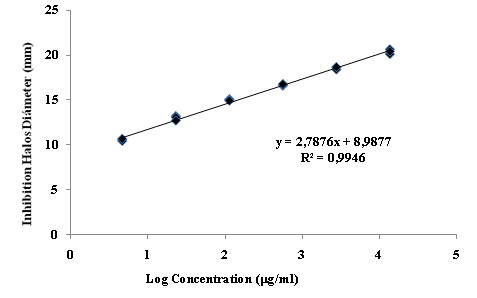
**Calibration curve with 10 concentration levels of Piperacillin/Tazobactam to determine the optimal test range**.

With Piperacillin/Tazobactam, our analysis demonstrated that the concentrations between C5 and C10 generated inhibition haloes of the appropriate diameter, so this range was evaluated (Figure 4). For concentrations between C1 and C4, the inhibition zones had diameters greater than 30 mm. Table 2 shows the range between C5 to C9 to be the most linear (R^2 ^= 0.9989).

**Table 2:** Evaluation of the range of concentrations of Piperacillin (USP standard)/Tazobactam

Concentration Range		Equation		
**From**	**To**	**Slope**	**Intercept**	**R^2^**
**C5**	**C9**	**2.5554**	**9.8253**	**0.9989**
C6	C10	2.7968	9.1638	0.992

#### Linearity

In Tables 3 and 4, antibiotic concentration correlates well with the diameter of the zone of inhibition.

**Table 3:** Evaluation of linearity for Meropenem and Piperacillin/Tazobactam

		Experimental t		Theoretical t	Decision
				
Test	HYPOTHESIS	Meropenem	Piperacillin		
**Slope**	H_0_: m = 0		2.684	2.16	Reject H_0_
	H_1_: m ≠ 0	7.677			
**Intercept**	H_0_: b = 0		18.025		Reject H_0_
	H_1_: b ≠ 0	47.901		2.16	
**Correlation**	H_0_: R = 0	58.41	23.381	2.16	Reject H_0_
	H_1_: R ≠ 0				

**Table 4:** Regression analysis by analysis of variance (ANOVA).

		Experimental t			
				
Test	HYPOTHESIS	Meropenem	Piperacillin	Theoretical t	Decision
	H_0_: There is no regression	3494.54	324.89	3.67	Reject H_0_
**Regression**	H_1_: There is regression				
	H_0_: There is no deviation				
**Linearity**	from linearity				
**Deviation**	H_1_: There is a deviation	0.3513	3.71	3.71	Accept H_0_
	from linearity				

From this point on, the selected concentrations will be designated C1 to C5 for clarity.

#### Precision

The reproducibility and between-days precision of our assays were evaluated in several ways. Reproducibility was studied by determining the coefficient of variation. This was less than 1%, which is acceptable for analytical assays in the pharmaceutical industry. The Cochran test was also applied, and the results showed that the variances at each concentration were equivalent. Finally, ANOVA for each concentration demonstrated no significant differences between two samples run on the same day (Table 5).

**Table 5:** ANOVA of the reproducibility of assays using Meropenem and Piperacillin/Tazobactam

Concentration	Experimental F		Theoretical F	Decision
			
	Meropenem	Piperacillin		
**C1**	2.9136	2.684	5.99	Accept H_0_
**C2**	3.9852	3.025	5.99	Accept H_0_
**C3**	1.1247	1.009	5.99	Accept H_0_
**C4**	0.4746	0.395	5.99	Accept H_0_
**C5**	0.4938	0.412	5.99	Accept H_0_

The between-days precision was also analyzed. ANOVA showed that for the antibiotics evaluated, the results of assays performed on different days did not differ significantly (Table 6).

**Table 6:** ANOVA of the between-days precision of assays using Meropenem and Piperacillin/Tazobactam

Concentration	Experimental F		Theoretical F	Decision
			
	Meropenem	Piperacillin		
**C1**	0.2747	0.6425	5.99	Accept H_0_
**C2**	0.0169	0.2342	5.99	Accept H_0_
**C3**	0.6447	0.4756	5.99	Accept H_0_
**C4**	0.8572	0.9325	5.99	Accept H_0_
**C5**	0.0712	0.123	5.99	Accept H_0_

#### Stability

The stability of each compound during the experimental period was verified. Solutions of Meropenem and Piperacillin/Tazobactam (1 mg/ml; USP Standard) were incubated at 37°C, and samples were taken after 0, 6, 12, 24, and 48 h and six days of incubation. The corresponding dilutions were then evaluated, and the results were plotted and compared to reveal any reduction in antibiotic activity (i.e., a decrease in the diameter of the zone of inhibition).

From the equation *y = mx + b*, where *y *represents the inhibition zone diameter and *x *represents the log of the concentration, changes in the *b *value indicate changes in activity. If there is no change in the intercept, the antibiotic is stable. If the *b *value decreases, this indicates instability or a loss of activity.

In the case of Meropenem, the solution showed a slight decrease in the intercept value after 24 h incubation (Table 7). From this result, it appears that the molecule remained stable during our assays.

**Table 7:** Stability of Meropenem and Piperacillin/Tazobactam in water for injection at 37°C

Time	Meropenem			Piperacillin/Tazobactam		
	
	Slope	Intercept	R^2^	Slope	Intercept	R^2^
0 hours	4.1977	16.556	0.9922	2.7549	9.2827	0.9969
6 hours	ND	ND	ND	2.717	14.247	0.9946
12 hours	ND	ND	ND	2.7162	16.39	0.9986
24 hours	4.4628	15.921	0.9976	2.6777	9.4456	0.9896
48 hours	4.1291	13.257	0.9847	3.2429	7.1086	0.9806
6 days	4.1659	5.671	0.9998	2.5459	7.0209	0.9603

ND: Not determined

Piperacillin/Tazobactam showed more interesting results. After 6 and 12 h the solution was more active, indicated by an increase in the intercept value (Table 7). In other assays under different conditions (incubation at 4°C or 25°C), the solutions showed the same trend (Table 8). High-performance liquid chromatography (HPLC) or mass spectrometry would be necessary to identify any structural or conformational change in the compound that could explain this activity increase. Most intriguingly, the activity returned to its initial levels after 24 h.

**Table 8:** Stability of Piperacillin/Tazobactam in water for injection at 4°C and 25°C

Time	4°C			25°C		
	
	Slope	Intercept	R^2^	Slope	Intercept	R^2^
0 hours	2.8549	9.2827	0.9969	2.7549	9.2827	0.9969
6 hours	2.8705	10.204	0.9949	2.7902	10.32	0.9892
12 hours	2.8234	13.45	0.9895	2.7247	13.355	0.9838
24 hours	2.8507	10.185	0.99	2.7927	9.4542	0.9908
48 hours	2.8802	8.9856	0.9955	2.8346	9.4962	0.9929
6 days	2.8158	9.4177	0.9987	2.8067	7.2892	0.9995

#### Specificity

Solutions of the antibiotics were incubated at 50°C. The Meropenem solution lost its activity after 22 days, meaning that it was the only molecule in solution responsible for the antimicrobial activity. The activity of Piperacillin/Tazobactam was lost after six days.

### Sample analysis

The samples were analyzed in two groups. The first was used when the assay was being validated, and the second to evaluate antimicrobial activity by determining the MIC, MLC and CC values for each antibiotic. The results were quantified using the statistical method described by Hewitt (1977). Table 9 shows the content of each antibiotic in the samples purchased, and in each case the values fulfill the criteria laid out by USP 29 NF 24 for intravenous Piperacillin and Meropenem: **"...Contents no less than 90% and no more than 120% of Piperacillin, calculated on anhydrous base of the quantity registered of Piperacillin" **and **"...Contents no less than 90% and no more than 120% of Meropenem, calculated on anhydrous base of the quantity registered of Meropenem."**

**Table 9:** Contents of the commercial samples

Samples	Piperacillin/Tazobactam		Meropenem	
	
	Group 1	Group 2	Group 1	Group 2
1	103.9	103.6	100.85	107.5
2	99.0	115.8	94.9	109.5
3	116.0	109.7	93.52	105.3
4	110.8	109.4	94.30	116.9
5	119.2	108.7	91.92	104.5
6	1156	105.3	97.75	105.9
7	110.6	110.5	94.02	107.2
8	113.9	111.3	94.64	115.7
9	114.7	104.2	90.76	107.2
10	116.2	107.9	92.89	103.3
11	111.2	116.8	93.32	105.7
12	112.4	117.6	95.28	107.1
13	114.6	114.3	95.29	102.5
14	116.2	108.9	92.86	103.4
15	108.2	110.1	93.43	106.2
16	114.1	108.5	93.84	104.5
17	117.9	112.6	94.22	105.1
18	110.3	104.9	97.32	106.7
19	114.8			105.8
20	111.9			107.1
21	109.9			

### Minimal inhibitory and lethal concentrations

**Figure 5:**
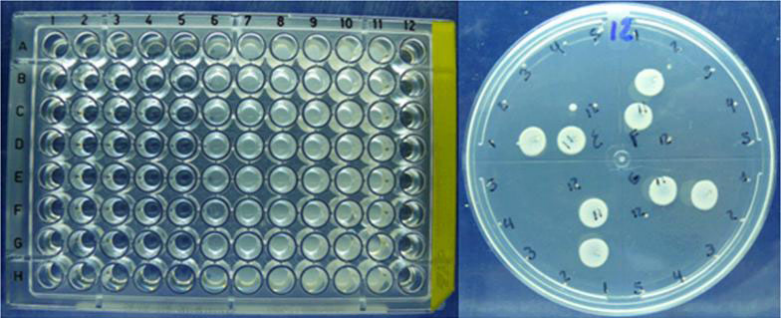
**MIC and MLC assay for Piperacillin/Tazobactam**.

Using previously described methods, the samples were analyzed in groups of seven per plate, each plate being inoculated with a single microorganism. The first row of the plate corresponded to the USP standard; the other seven rows contained the samples. Figure 5 shows the results for Piperacillin/Tazobactam. The plate shows the same performance for the standard as for the samples.

That is, growth was inhibited at the same concentration of each sample. After replication on to MH agar, there was no growth in concentrations C1 to C5 or C12, but there was growth in C6 to C11. This means that the antibiotic has an MLC but no MIC. The MLC is C5 for the USP standard and for all the samples. For all samples, using all microorganisms evaluated, the results showed the same performances for both molecules (Tables 10 and 11 include results for only some samples, as illustration).

**Table 10:** Determination of MIC and MLC for Meropenem

**Strain**	**MIC (μg/ml)**			**MLC (μg/ml)**		
	
	**Std**	**M1**	**M2**	**Std**	**M1**	**M2**
	
*Ps*. *aeruginosa *151	3.9	3.9	3.9	7.8	7.8	7.8
*Ps*. *aeruginosa *157	15.6	15.6	15.6	31.3	31.3	31.3
*Ps*. *aeruginosa *ATCC 25619	31.3	31.3	31.3	62.5	62.5	62.5
*Ps*. *aeruginosa *74	31.3	31.3	31.3	62.5	62.5	62.5
*Ps*. *aeruginosa *142	15.6	15.6	15.6	31.3	31.3	31.3
*A*. *baumanii *59	62.5	62.5	62.5	125	125	125
*A*. *baumanii *147	15.6	15.6	15.6	31.3	31.3	31.3
*A*. *baumanii *173	0.98	0.98	0.98	1.95	1.95	1.95
*A*. *baumanii *189	15.6	15.6	15.6	31.3	31.3	31.3
*E. coli *39	3.91	3.91	3.91	7.8	7.8	7.8
*E. coli *50	62.5	62.5	62.5	125	125	125
*E. coli *69	31.3	31.3	31.3	125	125	125
*E. coli *ATCC 13706	≥ 500	≥ 500	≥ 500	≥ 500	≥ 500	≥ 500
*S*. *faecalis*	≥ 500	≥ 500	≥ 500	≥ 500	≥ 500	≥ 500
*S*. *faecalis *ATCC 29212	31.3	31.3	31.3	62.5	62.5	62.5
*S*. *faecalis *ATCC 319623	31.3	31.3	31.3	62.5	62.5	62.5
*E*. *gallinarum*	15.6	15.6	15.6	31.3	31.3	31.3
*K. pneumoniae *1	62.5	62.5	62.5	125	125	125
*K. pneumoniae *43	250	250	250	≥ 500	≥ 500	≥ 500
*K. pneumoniae *63	31.3	31.3	31.3	125	125	125
*K. pneumoniae *65	15.6	15.6	15.6	31.3	31.3	31.3
*K. pneumoniae *207	3.9	3.9	3.9	7.8	7.8	7.8
*K. pneumoniae *ATCC10031	125	125	125	250	250	250

**Table 11:** Determination of MIC and MLC for Piperacillin/Tazobactam

Strain	MIC (μg/ml)			MLC (μg/ml)		
	
	Std	M1	M2	Std	M1	M2
*A*. *baumanii *(*A. b*.) 59	500	500	500	1000	1000	1000
*A*. *baumanii *139	500	500	500	1000	1000	1000
*A*. *baumanii *147	500	500	500	1000	1000	1000
*A*. *baumanii *173	Nd	Nd	Nd	125	125	125
*S*. *faecalis *(*S. f*.)	Nd	Nd	Nd	31.25	31.25	31.25
*S*. *faecalis *ATCC 29212	Nd	Nd	Nd	250	250	250
*S*. *faecalis *ATCC 319623	1000	1000	1000	>1000	>1000	>1000
*E. gallinarum *(*E. g*.)	3.95	3.95	3.95	7.81	7.81	7.81
*E. coli *(*E. c*.) 39	Nd	Nd	Nd	7.81	7.81	7.81
*E. coli *50	1000	1000	1000			
*E. coli *69	62.25	62.25	62.25	250	250	250
*K. pneumoniae *(*K. p.) *1	Nd	Nd	Nd	62.51	62.51	62.51
*K. pneumoniae *43	R	R	R	R	R	R
*K. pneumoniae *63	Nd	Nd	Nd	1000	1000	1000
*K. pneumoniae *65	Nd	Nd	Nd	500	500	500
*K. pneumoniae *207	R	R	R	R	R	R
*Ps*. *aeruginosa *(*P. a*.) 42	Nd	Nd	Nd	7.81	7.81	7.81
*Ps*. *aeruginosa *74	125	125	125	250	250	250
*Ps*. *aeruginosa *151	31.25	31.25	31.25	62.51	62.51	62.51
*Ps*. *aeruginosa *157	62.51	62.51	62.51	125	125	125
*St. aureus *(*S. a*.) *287*	Nd	Nd	Nd	7.81	7.81	7.81
*St. aureus 291*	31.25	31.25	31.25	62.51	62.51	62.51
*St. aureus 25923*	Nd	Nd	Nd	31.25	31.25	31.25

### Critical concentration (CC)

The CC is the minimal concentration that inhibits microorganism growth. It is reached at the limit of the inhibition halo. It is a measure of the microorganism's sensitivity and can be two to four times greater than the MIC, which is determined under different conditions. The CC can be defined mathematically as Ln(CC) = Ln(C_O_) - X^2^/DT_O_, where CC is the critical concentration, C_O _is the antibiotic concentration in the reservoir, X is the length of the crown (see above), D is the diffusion coefficient, and T_O _is the critical time. The intercept of a plot of Ln (C_O_) vs. X^2 ^is the Ln of CC [7].

**Figure 6:**
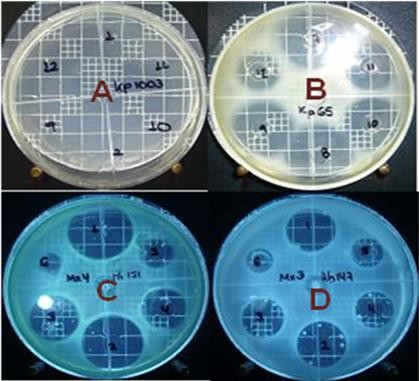
**Zones of growth inhibition produced by Meropenem against (A) *K. pneumoniae *ATCC 10031, (B) *K. pneumoniae *65, (C) *P. aeruginosa *151, and (D) *A. baumanii *147**.

**Figure 7:**
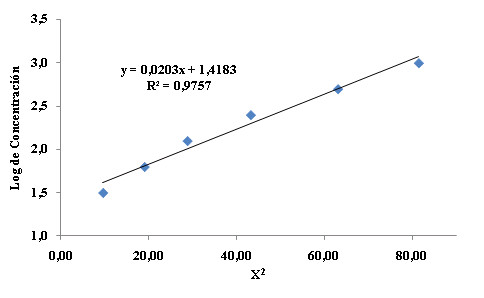
**Determination of critical concentration of Piperacillin/Tazobactam against *E. gallinarum***.

Figure 6 shows the different behaviors of the microorganisms tested with Meropenem and Piperacillin/Tazobactam standards. In Figures 6A and 6B, the inhibition haloes are very diffuse, whereas Figures 6C and 6D correspond to microorganisms with well-defined haloes. Some microorganisms exhibited growth of spontaneous mutants (e.g., Figure 6D), allowing a new means of comparing the performances of the products tested to be developed. A well-defined inhibition halo was the selection criterion for evaluating CC. For the Meropenem assay, *P. aeruginosa *151 and 157 and *A. baumanii *148 and *K. pneumoniae *were selected, and for Piperacillin/Tazobactam, the selected microorganisms were *E*. *faecalis*, *E*. *faecalis *ATCC 29212, *P*. *aeruginosa *42, 74 and 151 and *S. aureus *287. Figure 7 shows the correlation of X^2 ^with the log of antibiotic concentration. The equation is *y *= 0.0203*x *+ 1.4183, and *b *is therefore 1.4183. The CC is equivalent to antilog (1.4183), i.e., 26.2 μg/ml.

The CC values for the different Meropenem and Piperacillin/Tazobactam samples showed no significant differences, meaning that the products behaved in similar ways against the different microorganisms tested (Tables 12 and 13). On this basis, the generic products manufactured to meet all the quality standards applied to pharmaceutical products perform as well as the newest versions of those products.

**Table 12:** Critical concentrations for different samples of Meropenem against different microorganisms.

Sample	Critical Concentration (μg/ml)					
	
	*P. a*. 151	*P. a*. 157	*E. c*. 69	*E. c*. 50	*A. b*. 147	*K. b*. 63
**Standard**	3.253	0.437	0.512	0.439	1.533	0.107
**M1**	3.474	0.467	0.547	0.469	1.637	0.114
**M2**	3.461	0.465	0.545	0.467	1.631	0.114
**M3**	3.490	0.469	0.549	0.471	1.645	0.115
**M4**	3.660	0.492	0.576	0.494	1.725	0.120
**M5**	3.377	0.454	0.531	0.456	1.591	0.111
**M6**	3.403	0.457	0.536	0.459	1.604	0.112
**M7**	3.461	0.465	0.545	0.467	1.631	0.114
**M8**	3.692	0.496	0.581	0.498	1.740	0.121
**M9**	3.445	0.463	0.542	0.465	1.623	0.113
**M10**	3.390	0.455	0.534	0.457	1.597	0.111
**M11**	3.409	0.458	0.537	0.460	1.607	0.112
**M12**	3.445	0.463	0.542	0.465	1.623	0.113
**M13**	3.377	0.454	0.531	0.456	1.591	0.111
**M14**	3.338	0.448	0.525	0.450	1.573	0.110
**M15**	3.425	0.460	0.539	0.462	1.614	0.113
**M16**	3.373	0.453	0.531	0.455	1.590	0.111
**M17**	3.380	0.454	0.532	0.456	1.593	0.111
**M18**	3.419	0.459	0.538	0.461	1.611	0.112
**M19**	3.455	0.464	0.544	0.466	1.628	0.114
**M20**	3.435	0.461	0.541	0.464	1.619	0.113

**Table 13:** Critical concentrations of different samples of Piperacillin/Tazobactam against various microorganisms.

Sample	Critical Concentration (μg/ml)						
	
	***S. f***.	*S. f*. 29212	***E. g***.	*P. a *42	*P. a *74	*P. a*. 151	*S. a*. 287
Standard	16.069	29.648	26.182	23.714	24.210	17.458	35.400
M1	16.696	30.775	27.386	24.662	25.348	18.296	36.887
M2	18.399	33.918	29.847	27.200	27.745	19.955	40.604
M3	17.500	32.198	28.303	25.611	26.268	18.977	38.586
M4	17.451	32.228	28.512	25.658	26.244	18.977	38.444
M5	17.403	32.020	28.355	25.706	26.147	18.907	38.374
M6	16.809	31.042	27.439	24.876	25.324	18.296	37.170
M7	17.516	32.554	28.695	26.061	26.583	19.117	38.869
M8	18.014	33.325	29.559	26.749	27.261	19.588	39.648
M9	16.905	31.160	27.465	25.018	25.614	18.401	37.099
M10	17.098	31.694	27.936	25.255	25.760	18.663	37.949
M11	18.576	34.214	30.319	27.318	28.036	20.252	41.029
M12	19.107	35.311	31.340	28.290	28.810	20.793	42.197
M13	18.174	33.503	29.769	26.820	27.358	19.867	40.214
M14	17.500	32.376	28.721	25.777	26.244	19.047	38.728
M15	17.757	32.880	28.983	26.275	26.752	19.344	39.082
M16	17.355	31.991	28.303	25.540	26.026	18.855	38.232
M17	17.982	32.939	29.245	26.512	26.922	19.501	39.613
M18	16.937	31.427	27.727	24.947	25.639	18.418	37.276

In addition, the ratio between sample CC and standard CC is similar to the ratio of antibiotic contents. In others words, all samples perform the same with regard to their antimicrobial activities *in vitro *(Tables 14 and 15).

**Table 14:** Ratio of sample CC/standard CC for Meropenem

Sample	Sample CC/standard CC ratio						Ratio Median	Content
			
	*P. a*. 151	*P. a*. 157	*E. c*. 69	*E. c*. 50	*A. b*. 147	*K. b*. 63		
M1	106.8	106.9	106.8	106.8	106.8	106.5	106.8	107.5
M2	106.4	106.4	106.4	106.4	106.4	106.5	106.4	109.5
M3	107.3	107.3	107.2	107.3	107.3	107.5	112.5	105.3
M4	112.5	112.6	112.5	112.5	112.5	112.1	103.8	116.9
M5	103.8	103.9	103.7	103.9	103.8	103.7	104.6	104.5
M6	104.6	104.6	104.7	104.6	104.6	104.7	106.4	105.9
M7	106.4	106.4	106.4	106.4	106.4	106.5	113.4	107.2
M8	113.5	113.5	113.5	113.4	113.5	113.1	105.9	115.7
M9	105.9	105.9	105.9	105.9	105.9	105.6	104.1	107.2
M10	104.2	104.1	104.3	104.1	104.2	103.7	104.8	103.3
M11	104.8	104.8	104.9	104.8	104.8	104.7	105.9	105.7
M12	105.9	105.9	105.9	105.9	105.9	105.6	103.8	107.1
M13	103.8	103.9	103.7	103.9	103.8	103.7	102.6	102.5
M14	102.6	102.5	102.5	102.5	102.6	102.8	105.3	103.4
M15	105.3	105.3	105.3	105.2	105.3	105.6	103.7	106.2
M16	103.7	103.7	103.7	103.6	103.7	103.7	103.9	104.5
M17	103.9	103.9	103.9	103.9	103.9	103.7	105.0	105.1
M18	105.1	105.0	105.1	105.0	105.1	104.7	106.3	106.7
M19	106.2	106.2	106.3	106.2	106.2	106.5	105.6	105.8
M20	105.6	105.5	105.7	105.7	105.6	105.6	103.8	107.1

**Table 15:** Ratio of sample CC/standard CC for Piperacillin/Tazobactam

Sample	Sample CC/standard CC ratio							Ratio Median	Content
			
	***S. f***.	*S. f*. 29212	***E. g***.	*P. a*. 42	*P. a*. 74	*P. a*. 151	*S. a*. 287		
M1	103.9	103.8	104.6	104.0	104.7	104.8	104.2	104.3	103.6
M2	114.5	114.4	114.0	114.7	114.6	114.3	114.7	114.5	115.8
M3	108.9	108.6	108.1	108.0	108.5	108.7	109.0	108.5	109.7
M4	108.6	108.7	108.9	108.2	108.4	108.7	108.6	108.6	109.4
M5	108.3	108.0	108.3	108.4	108.0	108.3	108.4	108.3	108.7
M6	104.6	104.7	104.8	104.9	104.6	104.8	105.0	104.8	105.3
M7	109.0	109.8	109.6	109.9	109.8	109.5	109.8	109.6	110.5
M8	112.1	112.4	112.9	112.8	112.6	112.2	112.0	112.4	111.3
M9	105.2	105.1	104.9	105.5	105.8	105.4	104.8	105.3	104.2
M10	106.4	106.9	106.7	106.5	106.4	106.9	107.2	106.7	107.9
M11	115.6	115.4	115.8	115.2	115.8	116.0	115.9	115.7	116.8
M12	118.9	119.1	119.7	119.3	119.0	119.1	119.2	119.2	117.6
M13	113.1	113.0	113.7	113.1	113.0	113.8	113.6	113.3	114.3
M14	108.9	109.2	109.7	108.7	108.4	109.1	109.4	109.1	110.1
M15	110.5	110.9	110.7	110.8	110.5	110.8	110.4	110.7	110.1
M16	108.0	107.9	108.1	107.7	107.5	108.0	108.0	107.9	108.5
M17	111.9	111.1	111.7	111.8	111.2	111.7	111.9	111.6	112.6
M18	105.4	106.0	105.9	105.2	105.9	105.5	105.3	105.6	104.9

### Spontaneous mutants

**Figure 8:**
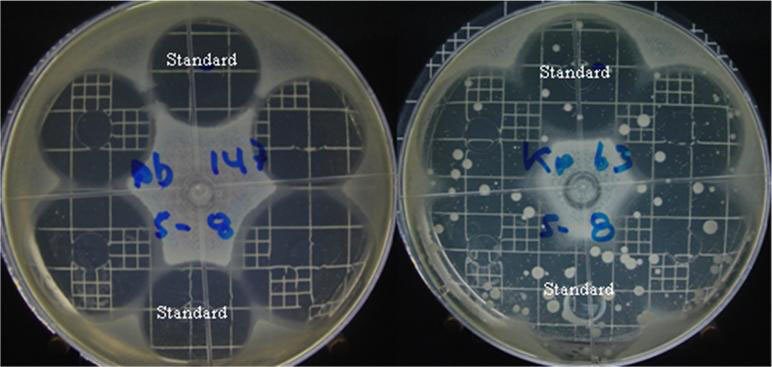
**Diffusion gel assay testing the production of spontaneous Meropenem-resistant mutants, with *A. baumanii *147 as a control strain and *K. pneumoniae *63 as a mutant-producing strain**.

It was noted in the previous assays that some strains produced spontaneous mutants (Figure 6), indicated by the appearance of colonies within the inhibition halo. Therefore, an assay to assess spontaneous mutation was developed with appropriate concentrations of antibiotics. Each experimental set-up included an agar plate inoculated with the test strain. Of the six reservoirs, two contained standard solutions and the other four contained sample solutions, as shown in Figure 8. The numbers of mutants produced by the standard and sample solutions were counted after incubation.

For Meropenem, the strains selected were *A. baumanii *147 as a control strain (showing no production of spontaneous mutants), *A. baumanii *189, *E. coli *39 and 69, and *K. pneumoniae *43 and 63. For Piperacillin/Tazobactam, the strains selected were *P. aeruginosa *151 as a control strain (showing no production of spontaneous mutants), *P. aeruginosa *74 and *A. baumanii *189. After statistical analysis, the results (Tables 16 and 17) showed no significant differences in the production of spontaneous mutants for any of the strains tested.

**Table 16:** Spontaneous mutant production in the diffusion gel assay for Meropenem

Sample	*A. b*. 189		*E. c*. 39		*E. c*. 69		*K. p*. 43		*K. p*. 63	
	
	Median	δ	Median	δ	Median	δ	Median	δ	Median	δ
Standard	25.333	1.366	205.333	5.354	43.333	2.582	43.333	2.582	203.167	2.137
M1	29.333	1.528	209.667	2.082	40.333	1.528	40.333	1.528	182.667	2.517
M2	22.667	1.528	207.333	1.528	46.333	3.055	46.333	3.055	198.667	4.041
M3	26.667	2.082	210.333	1.527	43.667	1.155	43.667	1.155	213.667	4.163
M4	22.000	2.000	208.000	2.000	44.667	1.528	44.667	1.528	200.000	6.245
M5	29.000	1.000	205.000	2.000	46.667	1.528	46.667	1.528	213.000	6.000
M6	29.333	2.082	209.667	1.155	41.333	1.528	41.333	1.528	196.667	7.095
M7	27.000	3.000	207.667	1.155	44.000	2.000	44.000	2.000	213.333	8.021
M8	23.000	2.000	207.667	1.155	45.667	1.528	45.667	1.528	202.333	6.110
M9	23.000	2.000	204.667	1.528	46.000	2.000	46.000	2.000	198.667	6.807
M10	29.000	2.000	202.333	2.517	47.667	1.528	47.667	1.528	213.667	8.083
M11	24.667	1.528	203.000	3.000	44.333	1.155	44.333	1.155	203.333	8.737
M12	26.667	1.528	210.000	1.000	40.000	1.000	40.000	1.000	188.333	4.163
M13	29.000	1.000	209.333	2.517	40.333	1.528	40.333	1.528	198.667	5.686
M14	24.667	1.155	202.667	3.786	44.333	1.528	44.333	1.528	203.667	6.110
M15	21.333	1.528	205.000	1.000	41.000	1.000	41.000	1.000	215.333	6.028
M16	28.333	1.155	208.667	1.528	44.667	1.528	44.667	1.528	199.000	4.000
M17	29.333	2.333	212.000	3.000	43.333	1.155	43.333	1.155	201.333	3.512
M18	23.667	1.155	205.000	1.000	46.000	1.000	46.000	1.000	209.000	9.165
M19	21.333	1.155	203.000	3.000	40.000	1.000	40.000	1.000	218.333	3.055
M20	23.333	2.516	204.667	2.082	45.667	1.155	45.667	1.155	210.333	7.095

F	8.715		3.571		6.076		4.683		7.294	
Prob.	0.000		0.000		0.000		0.000		0.000	
V C F	1.808		1.808		1.808		1.808		1.808	

**Table 17:** Spontaneous mutant production in the diffusion gel assay for Piperacillin/Tazobactam

Sample	*A. b*. 189		*P. a*. 54	
	
	Median	δ	Median	δ
Standard	125.17	1.472	110.00	9.381
M1	127.00	1.000	109.33	1.528
M9	123.67	2.517	104.67	1.528
M18	124.33	1.528	105.00	1.000
M6	125.67	1.528	109.67	1.155
M10	127.67	3.055	102.33	2.517
M16	128.33	1.528	109.67	0.577
M5	128.00	1.000	105.00	2.000
M14	124.33	1.155	101.67	2.082
M4	122.67	0.577	108.00	2.000
M3	125.67	2.082	111.00	1.732
M15	123.33	2.082	105.00	1.000
M7	127.67	1.528	107.67	1.155
M8	123.00	1.732	107.67	1.155
M17	129.33	5.859	108.67	1.528
M13	126.67	1.155	107.00	2.000
M2	123.33	1.528	107.33	1.528
M11	125.33	1.528	103.00	3.000
M12	125.67	2.517	110.00	1.000

F	2.657		1.898	
prob.	0.005		0.045	

## Discussion

The activity of an antibiotic can be assessed under controlled conditions by comparing the inhibition of growth of sensitive microorganisms by known concentrations of that antibiotic with a reference standard, producing meaningful results by well characterized methods [9-11]. The experiment to evaluate assay performance showed that it fulfilled the requirements (linearity, repeatability, precision). In the case of Meropenem, the best linearity was shown over the range 25 μg/ml to 1.5625 μg/ml, where the correlation was highest (R^2 ^= 0.9996). For Piperacillin/Tazobactam, the range from 62.5 μg/ml to 3.906 μg/ml was the most linear, with an R^2 ^value of 0.9989. The reproducibility and between-days precision of both assays had coefficients of variation less than 1%, and ANOVA showed no significant differences at any concentration. Antibiotic activity remained stable over the course of the assay at the selected temperature. Finally, the inhibition assay results were due only to the molecules evaluated. In conclusion, the assay was exact and accurate, with reproducible results.

Our results with Meropenem were generally similar to those of Mendez et al. (2005), but with some differences. Our work used *B. subtilis *ATCC 6633 as a test strain, while they selected *M. luteus *ATCC 9341. We used ten concentrations to ensure better statistical analysis; the other study only used three. However, Mendez et al. confirmed their results using HPLC. Similar results were reported by Zuluaga et al. (2009), who evaluated other antibiotics (Amikacin, Gentamicin and Vancomicyn). We conclude that antibiotics can be evaluated by established bioassays using an appropriate test microorganism and conditions, even though some evidence indicates high variability in bioassays.

Analysis of commercial versions of the antibiotics tested (innovator, trade mark and generic products) indicated that all the samples can be considered pharmaceutical equivalents because they fulfill the standards of the USP Pharmacopoeia (Table 9). Zuluaga et al. (2009) proposed a comparison of performances of all samples by linear correlation against the performances of an innovator to determine pharmaceutical equivalence. First, the content is determined in a comparison against USP standard (the so-called gold standard). In that study, the performance of all samples was similar to the standard, and the results were accurate and reproducible. Therefore, it is redundant to compare the behaviors of novel drugs against other samples. It is sufficient to determine whether they fulfill the standards of the appropriate regulatory agency because this can be shown using exact, accurate and reproducible methods.

It has been proposed that generic antibiotics behave differently from the innovator product against pathogenic microorganisms [4-6]. This is possible if the generic antibiotic does not fulfill quality standards for that pharmaceutical product (purity, content, etc.). For instance, contaminants in generic drugs may interfere with their antibiotic activities.

The MIC and MLC results obtained with different pathogenic strains showed no differences among samples (Tables 10 and 11). This is probably because the samples were pharmaceutical equivalents. We conclude that generic and novel products perform equally well. In other words, the generic products evaluated in this study fulfill the requirements to be considered for use in antimicrobial therapy.

We also designed an assay to determine critical concentrations using a few selected strains to confirm that all the generic products evaluated were effective in antimicrobial therapy. The results showed no significant differences among samples (Tables 12 and 13). Moreover, the ratios between the CC of the standard and those of the different samples were similar to their potency levels (Tables 14 and 15).

Along the same lines, an assay was designed to determine the production of spontaneous mutants in the diffusion gel assay. The results again showed that all the samples behaved similarly, leading us to conclude that none of the samples studied differs markedly in their antimicrobial activities. That is, generic and brand-name products that fulfill the international specifications for manufacturing pharmaceutical products behave similarly to novel products.

Our results are quite different from those of other studies [4-6]. Some of those studies were conducted using the newest product as a "standard of comparison", but the researchers did not take into account that a commercial product may have a range of content between 90% and 120%. Consequently, there will be great variability in the results with respect to the performance of the antibiotic. For instance, if the novel drug product has a hypothetical content of 120% relative to the declared content on the label and the generic product has a hypothetical content of 90%, then the effective content of the generic product would be 75% (90/120) of the novel drug. This could produce misleading results because although both products fulfill the content requirements, the first is at the upper limit and the second at the lower limit. Other assays have compared commercial antibiotic-discs with antibiotic-discs prepared from solutions of commercial products. Again, the exact content of each antibiotic-disc and the exact content of the product are unknown, so the results could be misinterpreted.

It has been proposed that generic antibiotics behave differently from the innovator product against pathogenic microorganisms [4-6]. This is possible if the generic antibiotic does not fulfill the quality standards for that pharmaceutical product (purity, content, etc.). For instance, contaminants in generic drugs may interfere with their antibiotic activities.

The MIC and MLC results obtained with different pathogenic strains showed no differences among samples (Tables 10 and 11). This is probably because the samples were pharmaceutical equivalents. We conclude that generic and novel products perform equally well. In other words, the generic products evaluated in this study fulfill the requirements to be considered for use in antimicrobial therapy.

## Conclusions

All the samples analyzed by standardized microbiological methods fulfill the requirements for content according to USP XXVII. They all show the same antimicrobial behavior because they have similar MIC, MLC and CC values and produce similar numbers of mutants.

## Abbreviations

MIC: Minimal Inhibitory Concentration; MLC: Minimal lethal concentration; CC: Critical Concentration; C1: Concentration 1; C2: Concentration 2; C10: Concentration 10; *A. b*.: *Acinetobacter baumanii*; *S. f*.: *Streptococcus faecalis*; *E. g*.: *Enterococcus gallinarum*; *E. c*.: *Escherichia coli*; *K. p*.: *Klebsiella pneumoniae*; *P. a*.: *Pseudomonas aeruginosa*; *S. a*.: *Staphylococcus aureus*; M1: Sample 1; M2: Sample 2; ...

## Competing interests

Diaz and Silva received financial support for lectures from Vitalis S. A. to participate in national scientific meetings in Colombia. The present study was a joint venture between the Science Faculty of National University of Colombia and Vitalis S.A., and was also financed by Vitalis S.A.

## Authors' contributions

ADLT and APH, both students at the National University of Colombia, jointly developed a process to validate the quantitative assay for Meropenem and Piperacillin/Tazobactam for their theses in Pharmaceutical Chemistry. MJA was the project administrator and contributed to article redaction. JAD and ES conceived the study, obtained necessary funding, designed and directed the execution and analysis of data, edited the manuscript and approved it for publication.

All the authors read and are in agreement with the whole all of article text.

## Pre-publication history

The pre-publication history for this paper can be accessed here:

http://www.biomedcentral.com/1472-6904/10/3/prepub
